# Advanced 3D In Vitro Liver Fibrosis Models: Spheroids, Organoids, and Liver-on-Chips

**DOI:** 10.3390/biomimetics10100639

**Published:** 2025-09-23

**Authors:** Jae Eun Lee, Yu-Jeong Lee, Jeong-Kee Yoon

**Affiliations:** 1 Department of Systems Biotechnology, Chung-Ang University, Anseong-si 17546, Republic of Korea; 2 Interdisciplinary Program in Bioengineering, College of Engineering, Seoul National University, Seoul 08826, Republic of Korea

**Keywords:** liver fibrosis, spheroids, organoids, liver-on-a-chip, preclinical model

## Abstract

Liver fibrosis (LF) is a progressive and increasingly prevalent condition, yet current therapeutic options remain limited. This underscores the growing demand for advanced three-dimensional (3D) preclinical models that better recapitulate the complex pathophysiology of human LF and overcome the limitations of conventional systems. Although a number of in vitro models have been proposed in recent years, many still rely on two-dimensional (2D) hepatocyte cultures, which fail to represent the multicellular interactions and spatial architecture of the fibrotic liver. In contrast, 3D in vitro models, including spheroids, organoids, and liver-on-a-chip (LoC) platforms, offer more physiologically relevant microenvironments, enabling improved disease modeling and patient-specific drug testing. In this review, we summarize current bioengineering strategies for constructing 3D LF models and highlight their advantages, limitations, and future directions for clinical translation.

## 1. Introduction

The liver plays a central role in maintaining systemic homeostasis by regulating metabolism, immune responses, digestion, detoxification, and vitamin storage. Every year, over two million deaths are attributed to liver diseases [1,2]. The etiologies of liver disease are diverse, including viral hepatitis (hepatitis B and C), alcohol-associated liver disease, and non-alcoholic fatty liver disease (NAFLD) [3]. Chronic liver damage resulting from these conditions initiates a wound-healing response, which can progress to liver fibrosis (LF), characterized by excessive accumulation of extracellular matrix (ECM) proteins within hepatic tissue [4]. As fibrosis advances, it may lead to cirrhosis, accompanied by architectural distortion and functional impairment of the liver [5,6].

Although a variety of in vivo and in vitro models have been developed to study LF, no single model fully replicates the complexity of human liver pathology. Animal models are commonly used due to their ability to capture systemic responses. However, they are associated with significant limitations, including high cost, long experimental timelines, species- and animal-specific physiological differences, and ethical concerns. Two-dimensional (2D) in vitro models, particularly hepatocyte cultures, offer a simpler and reproducible alternative but lack the structural and cellular complexity of the liver [7,8]. Specifically, 2D systems fail to reproduce the multicellular architecture, cell–matrix interactions, and spatial gradients that are critical to fibrotic progression and drug response [9,10]. In contrast, three-dimensional (3D) in vitro models provide a more physiologically relevant platform, with the potential to reduce reliance on animal testing and support patient-specific applications [11].

In this review, we summarize recent advances in the bioengineering strategies for modeling LF in vitro. We first examine 3D culture systems, including liver spheroids and organoids, and their capacity to recapitulate key fibrotic features. We then highlight organ-on-a-chip platforms that address the limitations of conventional 3D culture models by incorporating dynamic microenvironments and perfusable architecture. Finally, we discuss the current challenges associated with these platforms and provide perspectives on future directions to improve the physiological relevance and translational applicability of LF models for antifibrotic drug discovery. Special emphasis is placed on comparing the biological relevance, advantages, and limitations of each platform in the context of LF and NAFLD progression.

**Figure 1:**
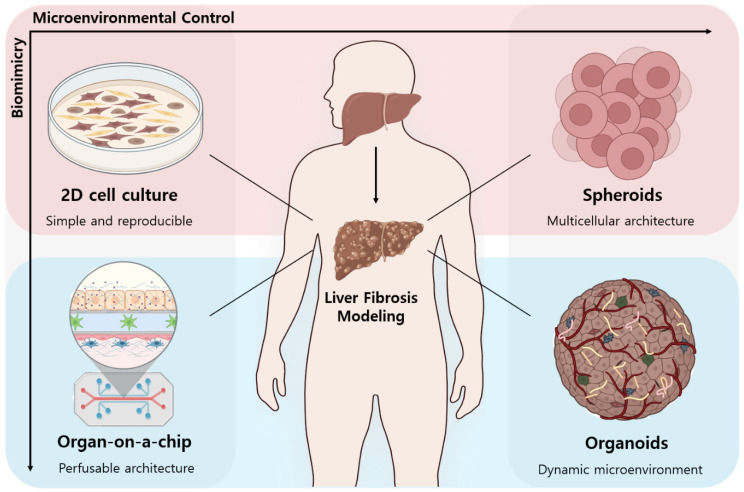
Schematic comparison of in vitro platforms for modeling liver fibrosis. Figure created with BioRender.com.

An overview is given of representative in vitro models used to study liver fibrosis (Figure 1). Each platform recapitulates distinct aspects of liver physiology and fibrosis progression with varying degrees of complexity: Two-dimensionally cultured cell lines (top left) remain the most widely adopted due to their simplicity, reproducibility, and compatibility with high-throughput assays; however, they lack the multicellular interactions and spatial organization of native liver tissue. Spheroids (top right), formed by the aggregation of hepatic or stromal cells, improve upon 2D cultures by enabling more realistic cell–cell contacts and partial three-dimensional structure. Organoids (bottom right), derived from stem or progenitor cells, exhibit self-organizing architecture and recapitulate key features of liver development and tissue function, offering greater physiological relevance. Finally, organ-on-a-chip systems (bottom left) integrate multiple hepatic cell types within perfusable microenvironments that allow for dynamic flow, biochemical gradients, and mechanical cues, thereby enabling advanced modeling of fibrogenesis in a highly biomimetic context. 

## 2. Pathogenesis of Liver Fibrosis

LF is an abnormal wound-healing response to chronic liver injury, characterized by the excessive deposition of ECM, which disrupts the normal hepatic structure [1]. This fibrotic process is a common pathological feature observed in most chronic liver diseases, including hepatitis B (HBV) or hepatitis C (HCV) viral infections, alcoholic liver disease, and non-alcoholic steatohepatitis (NASH) [12,13]. Regardless of the underlying etiology, fibrosis develops through a dynamic cascade, leading to cirrhosis and ultimately resulting in portal hypertension, hepatic failure, and hepatocellular carcinoma. Injured or dying hepatic cells release reactive oxygen species (ROS) and damage-associated molecular patterns (DAMPs), which activate resident immune cells and stimulate fibrogenic signaling [14].

Fibrosis progression is histologically graded into four stages: no fibrosis (F0), mild portal fibrosis (F1), periportal fibrosis with few septa (F2), bridging fibrous septa (F3), and cirrhosis (F4), which is an important factor for clinical outcomes. While early-stage fibrosis is potentially reversible upon removal of the injurious stimulus, advanced fibrosis and cirrhosis are generally considered irreversible and carcinogenic without a therapeutic intervention [5]. Indeed, understanding the mechanisms of liver fibrogenesis is critical for advancing antifibrotic therapeutic strategies.

Liver fibrosis (LF) is a wound-healing response to chronic liver damage characterized by excessive extracellular matrix (ECM) deposition. Upon hepatocellular injury, hepatocytes and cholangiocytes release reactive oxygen species (ROS) and damage-associated molecular patterns (DAMPs), which activate Kupffer cells and recruit monocyte-derived macrophages. These immune cells secrete pro-inflammatory and pro-fibrotic mediators such as TNF-α, IL-1β, TGF-β, and PDGF, initiating hepatic stellate cell (HSC) activation. Quiescent HSCs residing in the space of Disse lose vitamin A stores and transdifferentiate into proliferative, migratory, myofibroblast-like cells expressing α-SMA and COL1A1. Key intracellular signaling pathways include SMAD activation by TGF-β, MAPK/NF-κB-mediated cytokine expression by IL-1β and TNF-α, and Ras-Raf-MEK-ERK activation by PDGF. The resultant HSCs secrete large amounts of collagen I/III and TIMPs, contributing to increased matrix stiffness and sinusoidal capillarization. The right portion of the figure depicts fibrosis staging from F0 (normal) to F4 (cirrhosis), showing progressive ECM accumulation and architectural distortion. Crosstalk among immune cells, HSCs, endothelial cells, and platelets further amplifies the fibrogenic loop. Figure created with BioRender.com.

### 2.1. Hepatic Stellate Cell Activation and Transdifferentiation

**Figure 2:**
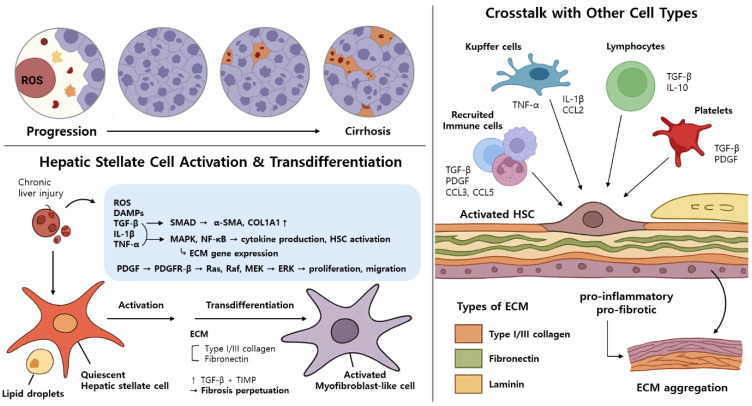
Overview of hepatic fibrosis progression and cellular signaling during chronic liver injury.

Hepatic stellate cells (HSCs) are the primary effector cells responsible for extracellular matrix production during LF (Figure 2). In healthy liver, HSCs reside in the perisinusoidal space of Disse and maintain a quiescent phenotype, characterized by vitamin A storage and the expression of PPARγ and glial fibrillary acidic protein (GFAP). Under chronic injury, persistent damage to hepatocytes and cholangiocytes induces cell death via apoptotic, necrotic, necroptotic, or autophagic pathways, triggering the release of inflammatory mediators, ROS, and DAMPs from the dying cells and the recruited immune cells. Especially, the inflammatory mediators, including tumor necrosis factor (TNF), ROS, interleukin-6 (IL-6), IL-1β, Hedgehog ligands, nucleotides, and transforming growth factor-β (TGF-β), initiate the transdifferentiation of quiescent HSCs into activated myofibroblast-like cells. [15].

Among the key mediators, TGF-β plays a central role in HSC activation as the most influential pro-fibrogenic cytokine secreted by various hepatic cell types. When TGF-β binds to type II receptor (TGFβR2) or type I receptor (TGFβR2 or ALK5) phosphorylates, this induces the phosphorylation of SMAD proteins, especially SMAD3 [16]. This SMAD3 activation facilitates the transcriptional upregulation of collagen type I and type III genes and contributes to fibrogenesis [17,18]. Beyond SMAD-dependent signaling, TGF-β also induces HSC activation via MAPK pathway components such as MAPK1, p38, and c-Jun *N*-terminal kinase (JNK) [19]. During the HSC activation process, quiescent HSCs undergo phenotypic change, which includes the downregulation of PPARγ, GFAP, and vitamin A storage, and upregulation of α-smooth muscle actin (α-SMA), collagen type I (COL1A1), and platelet-derived growth factor receptor-β (PDGFR-β) [15,20].

In addition to TGF-β signaling, platelet-derived growth factor (PDGF) is a most potent chemoattractant for HSCs’ proliferation, differentiation, and migration [21]. Activated HSCs overexpress PDGF, which binds to PDGFR-β and triggers the Ras-Raf-MEK-ERK signaling pathway. This cascade subsequently increases the expression of fibrogenic markers, such as type I collagen, α-SMA, and tissue inhibitors of metalloproteinases (TIMPs) [22]. Moreover, PDGF-mediated signaling activates the PI3K/Akt signaling pathway, which induces cytoskeletal reorganization and guides the migration of HSCs towards the injury site [23]. Therefore, activated HSCs accumulate at the injured sites and secrete large quantities of fibrillar collagens (type I and III), laminin, fibronectin, and other ECM components [5,14].

### 2.2. Crosstalk with Other Cell Types

The progression of LF involves complex intercellular communication beyond HSCs. Kupffer cells, the liver-resident macrophages, represent approximately 80–90% of all tissue macrophages in the human body. They serve as primary sensors of hepatocellular injury and are key regulators of inflammation, acting as major sources of inflammatory cytokines [24]. During chronic hepatic injury, Kupffer cells are activated by DAMPs (e.g., HMGB1 and DNA fragments) and gut-derived pathogen-associated molecules (e.g., LPS), primarily via Toll-like receptor signaling [25]. The activated Kupffer cells, together with liver sinusoidal endothelial cells and recruited immune cells, release pro-inflammatory and pro-fibrotic mediators including tumor necrosis factor-alpha (TNF-α), interleukin-1β (IL-1β), CCL2 (monocyte chemoattractant protein-1), and reactive oxygen species (ROS), which collectively amplify local inflammation and promote HSC activation [26,27,28]. In addition to Kupffer cells, monocyte-derived hepatic macrophages contribute to fibrogenesis through sustained secretion of cytokines and chemokines such as TGF-β, PDGF, TNF-α, IL-1β, CCL3, and CCL5 [19]. These macrophage-derived factors not only activate HSCs but also shape the immune landscape of the fibrotic liver [19].

Lymphocytes are one of the adaptive immune cells that can exert both pro-fibrotic and antifibrotic effects depending on their specific subtypes. In chronic liver injury, hepatocytes and non-parenchymal cells release pro-inflammatory mediators that facilitate lymphocyte recruitment to the injury site. Chemokines such as CXCL9, CXCL10, and CXCL11, secreted by HSCs and endothelial cells, interact with CXCR3 receptors on lymphocytes, promoting trans-endothelial migration [1]. Moreover, CD4^+^ T-cell differentiation into distinct T-helper subsets plays a key role in shaping their functional properties and cytokine profiles. Among them, Th2 cells promote fibrogenesis by upregulating pro-fibrotic genes and secreting immunoregulatory cytokines such as TGF-β and IL-10 [1,29].

Platelets also contribute to liver fibrogenesis by releasing a high amount of TGF-β and PDGFβ upon activation. They are the key HSC-activators that facilitate collagen overexpression [30]. These contribution of such platelet-derived signals have been repeatedly reported in the progression of fibrosis in various liver diseases including NASH, alcoholic liver disease, and viral hepatitis [31].

### 2.3. ECM Remodeling

The continuous accumulation of ECM proteins in the space of Disse progressively alters the liver microenvironment by elevating matrix rigidity and compactness. Activated HSCs and macrophages increase the expression of TIMPs, which suppress matrix degradation by matrix metalloproteinases (MMPs), resulting in a persistent imbalance between the production and breakdown of ECM and finally leading to ECM accumulation [14]. Subsequently, normal basement membrane components such as type IV collagen, laminin, and heparan sulfate proteoglycans are progressively replaced by interstitial collagens I and III. This shift increases matrix stiffness and disrupts the sinusoidal architecture, impairing hepatic function [13,32]. Furthermore, the downregulation of matricellular proteins like CCN3/NOV has been shown to promote fibrotic responses in HSCs [33]. Together, these pathological changes in matrix composition and turnover promote further capillarization and feed into a positive feedback loop that sustains fibrogenesis and leads to irreversible tissue remodeling [33].

## 3. Liver Fibrosis Modeling

### 3.1. Cell Lines

The selection of appropriate cell types is a critical determination in establishing physiologically relevant in vitro models of LF. Compared to in vivo models, cell-based models offer advantages in scalability, reproducibility, and cost-effectiveness [34]. In general, hepatic cell sources for fibrosis modeling can be classified into three types: tumor-derived cell lines, primary cell lines, and pluripotent stem cell (PSC)-derived cells.

Tumor-derived cell lines, such as HepG2 and Huh7, exhibit an indefinite proliferation capacity, while stably maintaining their hepatic phenotypes and compatibility with widely used high-throughput screening platforms [35]. However, they exhibit limited hepatic functionality, including low expression of cytochrome P450 enzymes and poor metabolic competence, which limits their utility in disease modeling and drug response assessment [36]. Primary cells, which include both hepatocytes and non-parenchymal cells (e.g., hepatic stellate cells, Kupffer cells, and liver sinusoidal endothelial cells), better preserve in vivo-like phenotypes and functional responses than tumor-derived cells [37]. One widely used one is the LX-2 cell line, an immortalized human HSC line that retains key fibrogenic properties and is able to be activated by TGF-β1 treatment to mimic fibrotic signaling. Nevertheless, primary cells suffer from a limited lifespan, donor variability, and susceptibility to rapid dedifferentiation under conventional 2D culture systems [38,39]. Pluripotent stem cells (PSCs), including embryonic stem cells (ESCs) and induced pluripotent stem cells (iPSCs), represent a promising alternative for generating hepatocyte-like cells or HSC-like cells in vitro [40]. The long-term self-renewal capacity of PSCs enables the generation of sufficient cell populations for repeated and reproducible studies [40]. In addition, the possibility of patient specificity from the donor makes PSCs suitable for disease stratification [40]. However, challenges remain in the form of inefficient and non-standardized differentiation protocols, high production costs, and residual epigenetic memory, which may hinder complete lineage conversion and reproducibility [40].

In addition to cellular components, the extracellular microenvironment also plays a key role in recapitulating key features of LF. A variety of natural and synthetic biomaterials, such as collagen, gelatin, hyaluronic acid, decellularized extracellular matrix (dECM), and synthetic polymers, are employed to provide structural and biochemical support to the cells. These materials enable the modulation of mechanical stiffness and cellular behavior, which is essential for modeling different stages of fibrogenesis [41]. Natural biomaterials derived from the ECM generally demonstrate strong biocompatibility and intrinsic bioactivity [42]. They provide native biochemical cues that support cell adhesion and tissue regeneration. In contrast, synthetic polymers lack such cell-interactive cues and frequently require surface interaction to avoid an immune response and enhance biological integration, but it is more feasible to control their mechanical properties [43]. Therefore, culturing hepatic cells onto an appropriate 2D substrate has been established as one of the most reproducible and high-throughput liver modeling methods. Nevertheless, the limitations in mimicking complex intercellular communications and inaccuracy due to the lack of 3D microenvironment have highlighted the need for advanced LF models such as spheroids, organoids, and liver-on-chips (LoCs).

### 3.2. Spheroids

**Figure 3:**
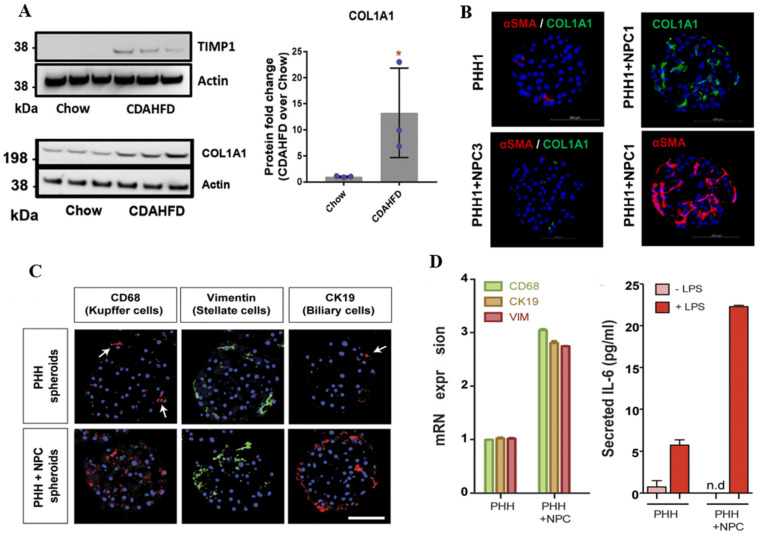
Characterization of liver fibrosis spheroid models using various cell combinations. (**A**) Reverse transcription polymerase chain reaction (RT-PCR) analysis of *TIMP1* and *COL1A1* mRNA levels in livers of mice fed a chow diet or choline-deficient, L-amino acid-defined, high-fat diet (CDAHFD) for 12 weeks. Data are presented as mean ± standard deviation (SD), *n* = 3 per group. Statistical significance is indicated by * *p* < 0.05. Data are shown as mean ± standard deviation (SD). (**B**) Immunofluorescence staining of spheroids composed of primary human hepatocytes (PHHs) in monoculture or co-cultured with non-parenchymal cells (NPCs), including hepatic stellate cells (HSCs), Kupffer cells (KCs), and liver sinusoidal endothelial cells. Co-cultured spheroids showed increased expression of collagen type I alpha 1 (COL1A1) and alpha-smooth muscle actin (αSMA). (**C**) PHH spheroids co-cultured with KCs, HSCs, and biliary epithelial cells were analyzed by immunofluorescence. Specific cell markers were used: CD68 (KCs), vimentin (HSCs), and cytokeratin 19 (CK19, biliary cells). White arrows indicate CD68^+^ Kupffer cells (left panel) and CK19^+^ biliary cells located at the spheroid periphery (right panel). (**D**) PHH spheroids co-cultured with KCs, HSCs, and biliary epithelial cells were analyzed by quantitative PCR. Co-cultures exhibited elevated interleukin-6 (IL-6) secretion compared to monocultures. (Reprinted with permission from ref. [44,47,48], Copyright 2024 Springer Nature).

Spheroid culture has been employed since the 1970s as a simple yet effective three-dimensional (3D) model, while 2D liver disease models fail to capture inter-individual variability or replicate the complex hepatic microenvironment. From a physiological standpoint, 2D culture relies on a planar attachment and lack of 3D ECM microenvironment. This leads to limited physiological relevance given the diminished polarity and altered cellular behavior. Rigid and static environments cause hepatocytes to dedifferentiate with loss of apico-basal polarity and bile canalicular organization. In contrast, 3D hepatocyte spheroids better preserve cell polarity than 2D monolayers by exhibiting well-formed tight junctions and microvilli-lined bile canalicular channels [44,45]. The physiological relevance of spheroid-based liver or its fibrogenic models is largely determined by their cellular composition, particularly the inclusion of human primary hepatocytes (PHHs) and non-parenchymal cells (NPCs), which collectively recapitulate the multicellular architecture of the liver. Such co-cultured spheroids incorporating PHH and NPCs—including hepatic stellate cells (HSCs), Kupffer cells (KCs), biliary epithelial cells, and liver sinusoidal endothelial cells—have enabled more physiologically relevant and patient-specific modeling of liver disease [46]. Hepatoma cell lines co-cultured with primary HSCs induced fibrotic activation by secreting fibrogenic factors, increasing the production of collagen type I alpha 1 (COL1A1) and TIMP metallopeptidase inhibitor 1 (TIMP1). These genes are strongly associated with fibrosis along with actin alpha 2 (ACTA2), a marker of activated HSCs. Such models also exhibit elevated expression of pro-inflammatory cytokines, demonstrating their utility for studying fibrogenic signaling (Figure 3A,B) [47,48]. In more complex models, PHH spheroids co-cultured with different types of NPCs have exhibited inflammatory phenotypes. For example, spheroids incorporating KCs, HSCs, and biliary cells showed strong expression of cell-specific markers, such as CD68 (Kupffer cells), vimentin (stellate cells), and cytokeratin 19 (CK19) (biliary cells), indicating that each cell type maintained its phenotypic identity and functional characteristics within the 3D microenvironment. This also suggests successful cellular integration and the potential for modeling liver-specific multicellular interactions. These co-cultures also exhibited significant upregulation of interleukin-6 (IL-6) secretion, indicating a pro-inflammatory state commonly associated with fibrosis [44] (Figure 3C,D). Furthermore, 3D hepatocyte spheroids have been shown to maintain higher levels of phase I and II metabolic enzyme expression and activity compared to conventional 2D monolayer cultures [49]. They also display greater sensitivity to hepatotoxic compounds and more physiologically relevant enzyme induction responses, like CYP3A4 induction, which are often not observed in 2D systems [50,51].

**Figure 4:**
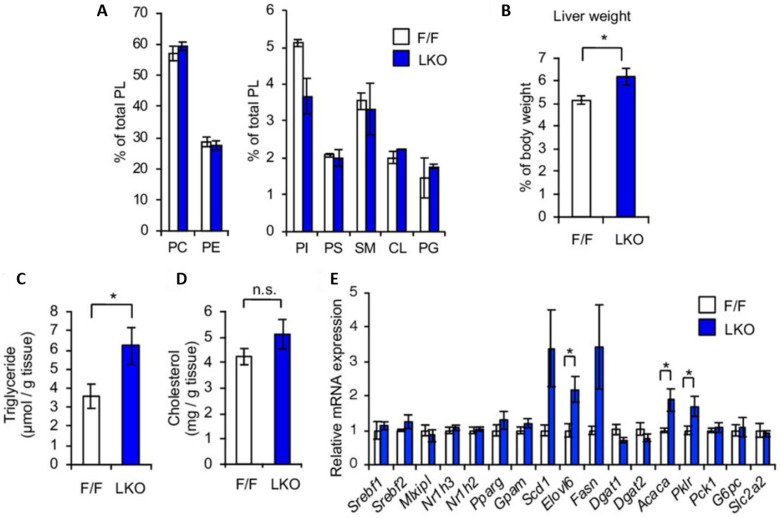
LPIAT1 knockout in hepatocytes promotes steatosis and lipid remodeling in liver tissue. (**A**) Phospholipid composition in liver tissues of wild-type (F/F) and liver-specific knockout (LKO) mice lacking lysophosphatidylinositol acyltransferase 1 (LPIAT1, also known as MB0AT7). Individual phospholipid species were quantified. (**B**) Liver weight was significantly increased in LKO mice compared to F/F controls. (**C**) Triglyceride (TG) levels were elevated in LKO livers, indicating increased neutral lipid accumulation. (**D**) Total hepatic cholesterol content showed no significant difference between groups. (**E**) Messenger RNA (mRNA) expression of genes related to fatty acid β-oxidation and gluconeogenesis remained unchanged, with the exception of pyruvate kinase liver and red blood cell isoform (*Pklr*), which was upregulated. Data are presented as mean ± standard error of the mean (SEM). Statistical significance was determined using an unpaired two-tailed Student’s *t*-test (* *p* < 0.05). (Reprinted with permission from ref. [52], Copyright 2021 BMJ).

In addition to multicellular complexity, spheroid platforms have also been utilized for modeling of the genetic components of NAFLD. For instance, silencing of MB0AT7 (also known as LPIAT1), a gene involved in phospholipid remodeling, led to increased lipid accumulation, collagen production, and HSC activation in Hepa1-6–HSC spheroids [52]. Additionally, knockdown of PNPLA3 I148M, a well-known NAFLD-associated genetic variant, resulted in enhanced lipid storage and collagen secretion, mimicking early fibrogenesis. These genetically modified spheroid models have been applied to high-throughput drug screening, leading to the identification of momelotinib, a JAK inhibitor, as a potential therapeutic for PNPLA3-related fibrosis [53] (Figure 4).

To fully emulate the fibrotic niche, it is also imperative to consider the role of ECM dynamics within the spheroid. The integration of biomaterial scaffolds has become increasingly central to achieve greater control over morphology, matrix stiffness, and cellular microenvironment. For instance, when fibroblast-like NIH3T3 was mixed with collagen to provide an extracellular matrix component in co-cultured spheroids of HUVECs and HepG2 cells, the tumor spheroids exhibited a markedly increased size with a noticeably reduced necrotic core [54]. Such structural and functional features are difficult to reproduce in 2D monolayer cultures, yet can be achieved in a 3D culture system through ECM modeling, thereby further supporting improved cell viability.

Beyond modeling fibrogenesis, spheroid-based systems have also demonstrated a superior sensitivity and dynamic range in chronic toxicity testing compared with 2D cultures, including the reproduction of the mitochondrial toxicity of fialuridine and cholestasis phenotypes under chlorpromazine treatment [44]. Moreover, multicellular spheroids incorporating hepatocytes, endothelial cells, macrophages, and HSCs significantly enhanced the predictive capacity for drug-induced liver injury, faithfully capturing steatosis, cholestasis, and fibrosis-related features [55]. These findings highlight the value of spheroids not only for mechanistic studies but also for therapeutic screening applications.

### 3.3. Organoids

Liver organoids have emerged as promising 3D in vitro platforms for modeling liver disease progression and evaluating antifibrotic therapies. Broadly, hepatic organoids can be classified into tissue stem cell-derived and pluripotent stem cell (PSC)-derived organoids, each offering distinct advantages in terms of physiological relevance and scalability. Earlier studies have utilized murine tissue-derived organoids, while more recent efforts have focused on human-derived organoids to study NAFLD and LF.

**Figure 5:**
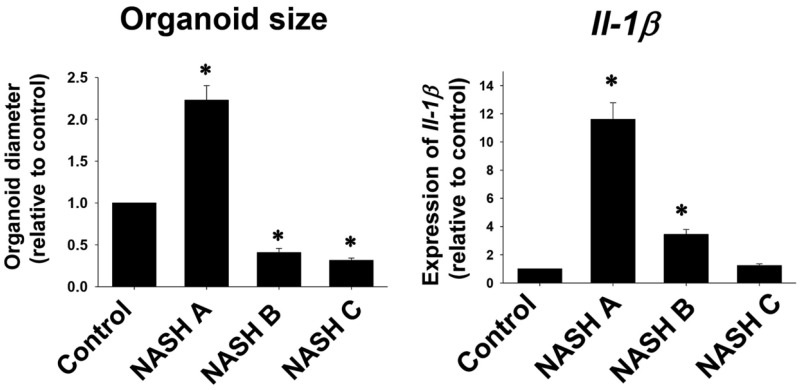
Fibrosis stage-specific characteristics in murine-derived hepatic organoids. Organoid size and expression of IL-1β were measured in three distinct non-alcoholic steatohepatitis (NASH) stages: NASH A, B, and C. Organoid size at day 5 (*n* = 4) was quantified using ImageJ and expressed as fold change relative to control (mean ± SEM; * *p* < 0.05). Il-1β expression was assessed by quantitative real-time PCR. Organoids derived from the NASH A stage displayed the largest diameter and highest IL-1β expression levels, suggesting a pro-inflammatory phenotype in early fibrogenesis. (Reprinted with permission from ref. [57], Copyright 2020 Elsevier).

To create murine tissue-derived organoids, wild-type or genetically modified mice were fed specialized or high-fat diets, after which liver tissue was harvested for cell isolation and cultured in hydrogel-based ECM supplemented with growth factors and stem cell medium [56]. Recent work has demonstrated that hepatic organoids can capture stage-specific fibrogenic responses. For example, organoids derived from hepatocytes and activated hepatic stellate cells (aHSCs) isolated from mice at three different stages of fibrosis revealed variations in lipid metabolism and genetic stability during NAFLD progression. Notably, early-stage organoids exhibited significantly larger diameters and higher expression of interleukin-1β (IL-1β), implicating early inflammation as a key driver of fibrogenesis (Figure 5) [57]. This model highlights the dynamic nature of cytokine expression over the course of disease progression, providing a temporal dimension to fibrosis studies. Murine tissue-derived organoids have also been employed to evaluate drug metabolism and detoxification. Pittol et al. (2020) examined the functional role of farnesoid X receptor (FXR) isoforms [58]; Sano et al. (2021) explored the oxidative and electrophilic stress responses via free amino acids (FAAs) and the Keap1–Nrf2 antioxidant defense pathway [59]; and Liu et al. (2021) assessed the therapeutic potential of uridine in alleviating lipotoxic stress [60]. Despite these advances, murine organoids show limited capacity to fully recapitulate the pathogenesis of human NAFLD, underscoring the need for human-derived organoid systems [61].

**Figure 6:**
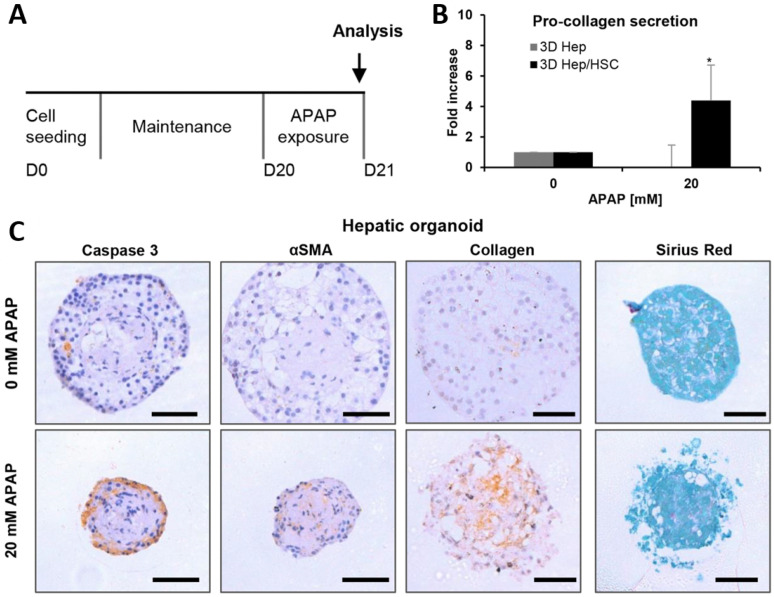
Drug-induced hepatic fibrogenesis in human 3D hepatic spheroids. (**A**) Schematic timeline of acetaminophen (APAP) exposure in 3D organoids composed of HepaRG hepatocytes (Hep) and primary human hepatic stellate cells (HSCs). Organoids were exposed to 20 mM APAP on day 20 and analyzed on day 21. (**B**) Pro-collagen secretion was significantly increased in Hep/HSC co-culture spheroids upon APAP exposure, compared to Hep-only spheroids. (*n* = 3 assays, pooled from six spheroids); significance indicated by * *p* < 0.05 versus solvent control (0 mM of APAP). (**C**) Immunohistochemical staining for cleaved caspase-3, alpha-smooth muscle actin (αSMA), collagen, and Sirius Red in hepatic organoids after 0 or 20 mM APAP treatment. Increased αSMA and collagen deposition were observed in the core region following APAP exposure. Hepatocytes were reduced at the organoid periphery, likely due to necrotic cell death. Scale bars: 50 µm. Scale bar = 50 μm; error bars denote SD. (Reprinted with permission from ref. [62], Copyright 2016 Elsevier).

In response, recent studies have established human liver organoids using various cell sources, including induced pluripotent stem cells (iPSCs), embryonic stem cells, hepatoblasts, and adult tissue-derived cells. One of the earliest human NAFLD organoid models utilized HepaRG hepatocytes (Hep) co-cultured with primary human HSCs in 96-well plates to form 3D spheroids. This model enabled the evaluation of hepatocellular sensitivity and HSC activation in response to compound exposure, thereby offering a platform for drug-induced LF testing (Figure 6) [57,62]. Importantly, the 3D spherical structure allows spatially controlled interactions between parenchymal and stromal cells, enabling more physiologically relevant fibrotic signaling than conventional 2D cultures [63,64,65]. More recently, hepatic stellate cells differentiated from iPSCs (iPSC-HSCs) have been introduced as an alternative to primary HSCs. These cells retain transcriptional and functional similarity to primary HSCs and maintain a quiescent phenotype when co-cultured in a spherical form with HepaRG cells. Upon exposure to hepatotoxic agents such as acetaminophen (APAP), iPSC-HSCs become activated, secreting pro-collagen, expressing α-smooth muscle actin (α-SMA, a marker of HSC activation), and increasing retinol storage as a fibrotic mediator. This platform recapitulates the wound-healing response triggered by hepatocyte injury and provides a tractable system for studying HSC activation and screening antifibrotic compounds [66].

**Figure 7:**
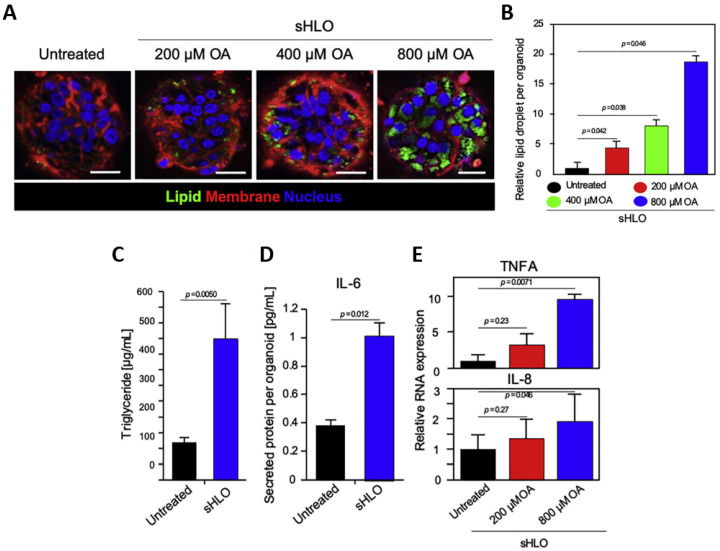
Lipotoxic injury and inflammatory responses in multicellular human liver organoids (HLOs). (**A**) Confocal microscopy images of HLOs exposed to increasing concentrations of oleic acid (OA: 200–800 µM) revealed dose-dependent accumulation of intracellular lipid droplets, visualized by lipid, membrane, and nuclear staining. (**B**) Quantitative analysis of lipid droplet numbers per organoid confirmed significant increases at higher OA concentrations. (**C**) Triglyceride levels, measured by enzyme-linked immunosorbent assay (ELISA), were significantly elevated in OA-treated HLOs. (**D**) Interleukin-6 (IL-6) secretion was increased in OA-treated organoids compared to untreated controls. (**E**) Relative RNA expression of tumor necrosis factor-alpha (TNFA) and interleukin-8 (IL-8) was upregulated in a dose-dependent manner following OA exposure. (Reprinted with permission from ref. [67], Copyright 2019 Elsevier).

To more closely mimic the multicellular architecture of human liver tissue, recent studies have developed organoids comprising mesenchymal and epithelial lineages derived from PSCs. These multicellular human liver organoids (HLOs) include hepatocytes, stellate cells, and Kupffer-like macrophages and reflect key features of in vivo liver physiology. Upon exposure to free fatty acids (FFAs) such as oleic acid (OA), these HLOs display hallmark features of steatohepatitis, including lipid accumulation (i.e., steatosis), inflammation, and extracellular matrix deposition (i.e., fibrosis). Functional assays revealed increased triglyceride levels, interleukin-6 (IL-6) secretion, and upregulation of pro-inflammatory cytokines such as tumor necrosis factor-alpha (TNFA) and interleukin-8 (IL-8), confirming disease-relevant activation pathways (Figure 7) [67]. In addition to molecular assays, organ-level characteristics such as stiffness or hardness can be analyzed to provide a quantitative readout for fibrosis severity.

In addition to developmental and disease modeling, organoid platforms are increasingly applied for pharmacological evaluation. For instance, Wu et al. established PSC-derived liver organoids stimulated with fibrogenic agents such as TGF-β, LPS, and methotrexate, which reproduced hallmark fibrosis phenotypes including collagen deposition, HSC activation, and inflammatory responses. When exposed to hepatotoxicants and antifibrotic compounds, these organoids exhibited a downregulation of fibrosis markers and suppression of myofibroblast-associated gene expression [68]. Such results underscore the potential of liver organoids as preclinical platforms for antifibrotic drug screening.

### 3.4. Liver-on-Chips

While spheroids and organoids have demonstrated strong potential as 3D platforms for modeling NAFLD and LF, they are inherently limited by passive nutrient and oxygen diffusion, poor waste clearance, and a lack of physiologically relevant shear stress [69]. To overcome these challenges and better simulate microscale tissue dynamics, LoC platforms have emerged as next-generation in vitro models [70]. The hallmark of LoC systems is the incorporation of microfluidic channels that allow for controlled medium flow, biomolecular gradients, and real-time oxygenation.

**Figure 8:**
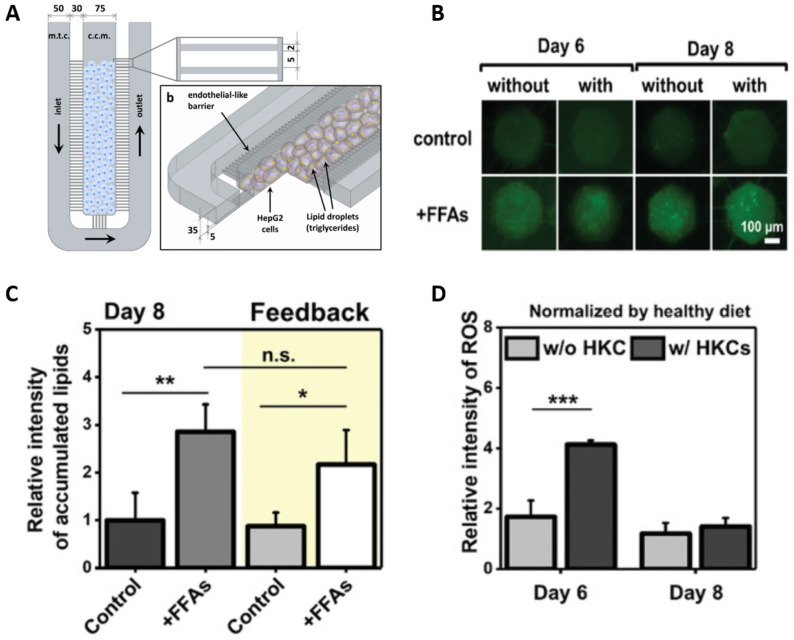
(**A**) Schematic representation of the first fibrosis LoC platform. Parallel microchannels mimic the endothelial barrier, allowing exchanges between the cells and the flowing culture medium but protecting HepG2 from the shear stress. (Reprinted with permission from ref. [71], Copyright 2016 PLoS One.) (**B**) Human umbilical vein endothelial cell (HUVEC)-derived spheroids with GelMa-encapsulated HepG2 with KCs. The spheroids with and without the inclusion of human KC (HKCs) showed a difference on days 6 and 8. The addition of HKC leads to an increase in lipid accumulation in the spheroids for the chip. (**C**,**D**) Inclusion of HKC also displayed a higher level of ROS on day 6 compared to those without it; however, on day 8, there was no significant ROS difference between samples with or without HKCs. Overall, including HKCs in liver spheroids enables a better in vitro system. Statistical significance was determined by ANOVA (* *p* < 0.05, ** *p* < 0.005, *** *p* < 0.0005 ). Error bars represent SD. (Reprinted with permission from ref. [72], Copyright 2019 Wiley).

The first LoC model employed HepG2 cells cultured in parallel microchannels to replicate endothelial barriers and assess free fatty acid (FFA)-induced steatosis (Figure 8) [71]. Subsequent studies incorporated multiple cell types, enabling closer mimicry of the hepatic microenvironment and more accurate modeling of disease progression. For instance, a co-culture of GelMA (gelatin methacryloyl)-encapsulated HepG2 cells and human umbilical vein endothelial cells (HUVECs) enabled a visualization of NAFLD-related changes in cell viability and lipid accumulation [72]. The use of GelMA provides a biocompatible matrix resembling native ECM and supports cell adhesion, proliferation, and tissue remodeling. When KCs were added to this model, elevated levels of ROS and pro-inflammatory cytokines were observed, suggesting a fibrotic shift in the microenvironment.

**Figure 9:**
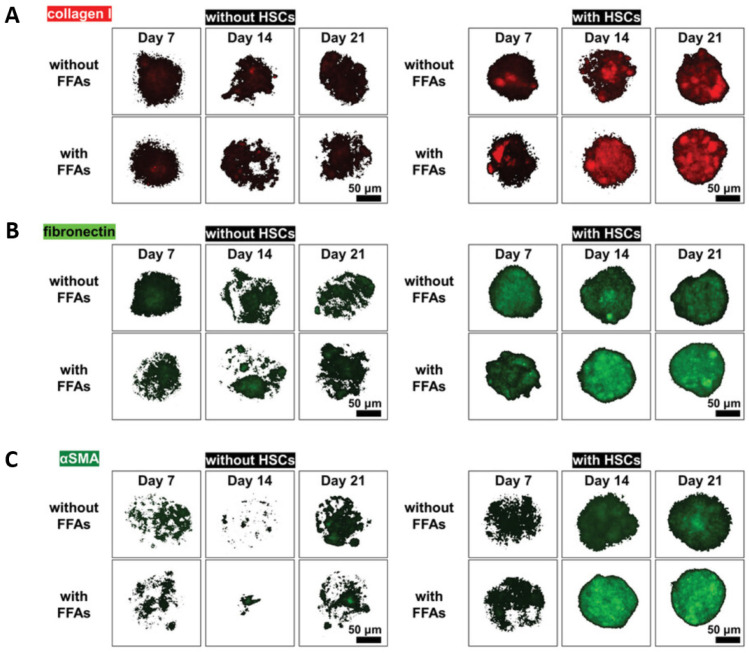
Effects of the presence of HSCs on hepatic fibrosis progression of BE-MLMs. (**A**) Collagen I, (**B**) fibronectin, and (**C**) αSMA in BE-MLM levels detected, with or without the HSC existence and with or without the FFA, showed much higher expression signals of collagen I, fibronectin, and αSMA than other groups without HSCs. (Reprinted with permission from ref. [77], Copyright 2021 Wiley).

While HepG2 cells remain commonly used in LoC platforms due to ease of culture, their limited hepatic functionality has led to the adoption of more physiologically relevant alternatives. Instead of HepG2, PHHs enabled biotransformation studies due to their in vivo-like enzyme activity, but are limited by availability [73]. Thus, HepaRG cells have been increasingly adopted as an alternative by offering a balance of accessibility and metabolic competence, such as enhanced cytochrome P450 enzyme activity, greater sensitivity to toxicants, and improved steatosis reversibility compared to HepG2 [74,75,76]. A recent study employed HepaRG instead of HepG2 in a LoC platform, and it reported increased secretion of inflammatory cytokines and improved modeling of steatohepatitis [72]. Furthermore, when HSCs were incorporated into this platform along with KCs and HUVECs, significant upregulation of collagen I, fibronectin, and α-SMA was observed following FFA treatment. This confirms the pivotal role of HSCs in driving fibrogenesis within engineered liver tissue (Figure 9) [77].

**Figure 10:**
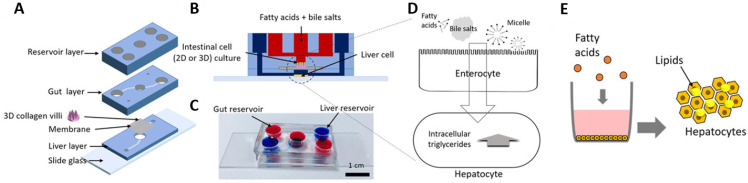
(**A**) A schematic view of a gut–liver chip. The membrane separates the gut and liver layers. (**B**) Side view of gut–liver chip. (**C**) An image of the gut–liver chip. The blue channel refers to the liver compartment and the red refers to a gut compartment. (**D**) Overview of the absorption mechanism in gut cells and liver cells in the gut–liver chip. (**E**) A schematic illustration of the addition of fatty acids to a well where hepatocytes are being cultured and quantified. (Reprinted with permission from ref. [79], Copyright 2018 Wiley).

Expanding beyond liver-specific modeling, multi-organ-on-chip systems, also known as body-on-a-chip platforms, have been developed to emulate physiological inter-organ communication. These models enable predictions of systemic drug responses and reduce failure rates in drug development. One such gut-liver-on-chip platform connected intestinal (Caco-2) and hepatic (HepG2 or PHH) compartments to simulate enterohepatic circulation and dietary lipid absorption. A porous membrane enabled metabolite exchange and compartmental separation. In this system, the short-chain fatty acid butyrate, known to enhance gut barrier integrity, was shown to suppress lipid accumulation in hepatocytes—a phenomenon not observed in isolated liver models (Figure 10) [78]. This result underscores the importance of tissue–tissue interactions in accurately modeling NAFLD pathogenesis and drug response. Overall, LoC technology offers a scalable, dynamic, and physiologically relevant platform that overcomes many limitations of static 3D cultures, enabling precise monitoring of hepatic function, fibrosis progression, and therapeutic response under flow conditions.

## 4. Conclusions and Future Perspectives

**Figure 11:**
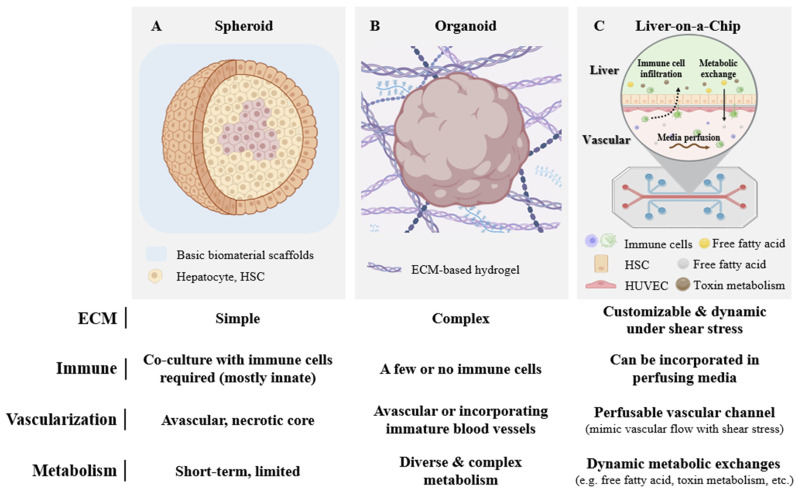
Comparison of structural and functional complexity in liver-mimetic platforms. Created with BioRender.com.

Recent advances in in vitro LF modeling have led to the development of diverse 3D culture systems that aim to improve physiological relevance. The selection of an appropriate platform remains a key consideration for both developmental studies and drug screening (Figure 11, Table 1 and Table 2).

Liver spheroids are widely used 3D culture systems due to their simplicity, cost-effectiveness, mass productivity, and relative ease of handling, while offering human organ-like complexity through self-organization from tissue- or iPSC-derived cells. Spheroids can be generated within a few days, making them particularly well-suited for high-throughput studies, while organoids provide human organ-like complexity useful for mechanistic insights. Spheroids also provide an accessible and cost-effective platform that enables high-throughput assessment of fibrogenic extracellular matrix deposition. However, their restricted structural organization limits their physiological relevance. Unlike spheroids, organoids have self-organizing abilities that allows complex ECM remodeling. Moreover, both spheroids and organoids provide accessible 3D culture models that better recapitulate hepatocyte functions than traditional 2D monolayers, making them valuable tools for basic and translational liver research. Within immune studies, spheroids and organoids can partially model interactions with immune cells by co-culturing with innate immune cells like Kupffer cells, but they do not provide systemic complexity needed for adaptive immune responses. Nonetheless, both systems are generally restricted by their growth in non-adhesive plates or hydrogels, resulting in limited structural guidance and reproducibility. Both spheroids and organoids lack vascularization and often exceed diffusion limits, leading to central necrosis in structures over 200 μm in diameter due to limited oxygen, nutrient, and waste transport [80,81]. Moreover, the absence of spatial organization hinders physiological relevance, especially in modeling hepatic cord-like structures or hepatocyte polarization [78]. Organoids typically rely on Matrigel, a xenogeneic matrix with undefined composition, resulting in batch-to-batch variability and compromised reproducibility [82]. These systems also remain limited in long-term culture stability, reproducibility, and scalability, highlighting the need for optimized biomaterials and automated bioprocessing.

LoCs provide a dynamic culture platform that addresses the limitations of spheroids or organoids by more accurately mimicking the hepatic microenvironment. LoCs consist of a controllable microfluidic perfusing system, mimicking vascular flow, gradients with nutrient exchange, and mechanical cues such as fluid shear stress, which are absent from spheroid or organoid models, and thereby provide the capacity to investigate ECM remodeling with precision under dynamic conditions [83,84]. These platforms also incorporate multiple types of cells, selected and combined from hepatocytes, endothelial cells, and immune cells such as KCs, which have improved the physiological accuracy of NAFLD and LF models. However, difficulties in co-culture with immune cells remain as a major limitation of LoCs despite their central role in NAFLD pathogenesis [78]. In addition, the complexity of microfluidic systems requires high expertise among users, adsorption of biomolecules to the polymer frame leads to inaccuracy of quantification, and the numerous different models require standardization in their widespread use in industrial screening workflows [85,86].

Looking ahead, advances in cell sourcing such as iPSC-derived hepatocytes or hepatic HSCs offer promising avenues for patient-specific and genetically stratified LF modeling. iPSC-derived liver organoids can also be a promising tool for developmental studies and screening of personalized medicine, but they still have limited application in disease modeling. To further expand modeling capacity, multi-organoid systems (i.e., assembloids) or multi-organ-on-a-chip systems (i.e., body-on-a-chips) are being explored as strategies to overcome the inability of current models to recapitulate systemic responses such as adaptive immune systems. Such multi-organ approaches, such as gut–liver systems, enable the study of inter-organ communication and metabolite transport, better expanding the physiological relevance of these platforms. To further increase predictive power, future studies may benefit from incorporating advanced analytic technologies such as spatial transcriptomics, single-cell multi-omics, and high-resolution imaging. In addition, coupling these datasets with machine learning and physiologically based pharmacokinetic modeling will enable predictive modeling and patient stratification. Recently, the combination of organ-on-a-chip platforms with organoids enabled synergistic effects, whereby organoids provide human organ-like complexity and organ-on-a-chips recreate the surrounding microenvironment to enhance homogeneity and reproducibility. This so-called organoid-on-a-chip hybrid approach offers greater physiological relevance than either platform alone, emerging as a promising tool for clinical translation.

Finally, the FDA is stepping into a new era by acknowledging non-animal tools called New Approach Methodologies (NAMs) as viable alternatives in preclinical drug testing. Their 2025 strategic roadmap highlights organ-on-chips, along with computational models and human-derived in vitro assays, as integral components of future safety and efficacy evaluations. Notably, through its Innovative Science and Technology Approaches for New Drugs (ISTAND) pilot program, the FDA has formally accepted a liver-based microphysiological system into its Drug Development Tool (DDT) qualification pipeline, underscoring the advancing regulatory readiness of such 3D in vitro platforms for integration into standardized drug development protocols. Alongside the FDA, the European Medicines Agency (EMA) is advancing initiatives to incorporate microphysiological systems into regulatory science, while actively continuing procedures for qualification and standards for acceptance. However, the key barriers to industrial adoption remain, including high initial manufacturing costs, batch-to-batch variability, and the need for fully GMP-compliant large-scale production systems. The collective progress highlighted in this review provides the foundation for building upon the potentially established regulatory frameworks for LF models. Such standardized guidelines and advancing technologies will therefore be essential to translate these experimental platforms into clinically relevant and industrially deployable tools (Table 2).

**Table 2:** Summary of advantages and disadvantages of liver fibrosis models.

Platforms	Advantages	Disadvantages	References
Animal model	(i). Vascularized tissue model (ii). Biobanking is feasible (at the cellular level) (iii). Systemic observation of the hepatic condition	(i). Unsuitable for high-throughput screening (ii). Unsuitable for organogenesis modeling (iii). Time-consuming (iv). Ethical issues (v). High heterogeneity	[87]
Monolayer	(i). Easily manipulated (ii). Low cost (iii). Good reproducibility (iv). High throughput	(i). Unable to recapitulate in vivo-like cellular morphology and 3D microenvironment (ii). Loss of cell polarity (iii). Poor functionality	[88,89]
Spheroids	(i). Simple, cost-effective, and easy to establish (ii). High scalability and reproducibility, suitable for high-throughput drug screening (iii). Maintains cell–cell interactions and partial ECM deposition	(i). Static culture, inefficient nutrient/waste transport (ii). Limited size control; necrotic core formation (iii). Lack of vasculature (iv). Limited immune activation without co-culture	[86]
Organoids	(i). Self-organized 3D liver-like structures (tissue- or iPSC-derived) (ii). Captures aspects of ECM remodeling and biliary structures (iii). Potential for patient-specific and genetically stratified modeling (iv). Allows longer-term maintenance compared to spheroids	(i). Dependent on Matrigel (batch variability, xenogeneic origin) (ii). Less scalable and reproducible for HTS (iii). Often avascular; immature vascularization (iv). Limited immune system recapitulation; culture stability challenges (v). Limited scalability and reproducibility	[82,86]
Liver-on-chips	(i). Dynamic microenvironment with microfluidic technology (ii). Suitable for co-culture, 3D culture, and integration of microtissues (iii). Improved liver-specific functionality (iv). Spatial control of the structure (v). High throughput and low cost (vi). Systemic observation (multi-organ-on-chips) (vii). Promising tools for translational application (viii). Adaptable for industrial drug screening	(i). Requirements of expertise for operation (ii). External devices required (e.g., pump) (iii). Non-specific adsorption of biomolecules to biomaterials (iv). Absence of standardized protocol (v). High diversity of chip designs limiting reproducibility	[85,86,90,91,92,93,94,95,96,97]

## Figures and Tables

**Table 1:** Comparative overview of 3D platforms for in vitro liver fibrosis modeling. ↑ represents elevated levels compared to the normal group.

Platforms	Cell Sources	Method to Induce Fibrosis	Methodological Strengths	Observations	Ref
Spheroid	PHH + HSCs	TGF-β1 stimulation	Enables multicellular fibrogenic signaling	↑COL1A1, TIMP1, ACTA2 (fibrosis markers)	[47]
	PHH + NPCs (KCs, biliary cells)	Cytokine exposure	Maintains cell-specific phenotypes in 3D	↑IL-6 secretion; preserved CD68, VIM, CK19	[48]
	Hepa1-6 + HSCs	MB0AT7 silencing	Captures genetic drivers of NAFLD	↑lipid accumulation, ↑collagen production	[52]
	Hepa1-6 + HSCs	PNPLA3 I148M knockdown	Models NAFLD-associated genetic variants	↑lipid storage, ↑collagen secretion	[53]
	HepG2 + HUVEC + NIH3T3 (with collagen)	ECM scaffold modulation	Provides ECM-based microenvironmental control	Reduced necrotic core; enlarged spheroid size	[54]
Organoid	Murine hepatocytes + activated HSCs	High-fat diet (in vivo) → organoid culture	Captures stage-specific fibrogenic responses	Early organoids: ↑IL-1β; stage-dependent lipid metabolism changes	[57]
	Murine tissue-derived organoids	Oxidative/electrophilic stress (FAAs)	Captures metabolic/ detox pathways	FXR isoform analysis	[58]
	Murine tissue-derived organoids	Oxidative/electrophilic stress (methionine and tyrosine metabolism)	Models stress responses during NAFLD progression	Functional detoxification assays	[59]
	Murine tissue-derived organoids	Lipotoxic/oxidative stress (Keap1–Nrf2 antioxidant pathway)	Enables antioxidant pathway analysis	Identified uridine effect in alleviating lipotoxic stress	[60]
	Human HepaRG + primary HSCs	Compound exposure	Provides parenchymal–stromal interaction	HSC activation under hepatocellular injury	[61]
	iPSC-derived HSCs + HepaRG	APAP (acetaminophen) exposure	Models hepatocyte injury-driven HSC activation cascade	↑pro-collagen, ↑α-SMA, ↑retinol storage	[65]
Liver-on-a-chip	HepG2 in parallel microchannels	FFA perfusion	Provides endothelial barrier mimicry under flow	Modeled FFA-induced steatosis	[70]
	GelMA-encapsulated HepG2 + HUVEC	FFA exposure	ECM-like GelMA supports adhesion and remodeling	Lipid accumulation; ↑ROS and ↑pro-inflammatory cytokines (with KCs)	[71]
	PHHs	Drug metabolism assays	Provides in vivo-like enzyme activity	Improved biotransformation and toxicant sensitivity	[72]
	HepaRG instead of HepG2	FFA exposure	Enhanced metabolic competence (↑CYP activity)	↑inflammatory cytokine secretion; improved steatohepatitis modeling	[73]
	HepaRG + HSCs + KCs + HUVECs	FFA treatment	Integrates multicell crosstalk under perfusion	↑collagen I, fibronectin, α-SMA expression (fibrogenesis recapitulated)	[76]

## Data Availability

No new data were created or analyzed in this study.
